# Use of Dietary Supplements among Professional Athletes in Saudi Arabia

**DOI:** 10.1155/2013/245349

**Published:** 2013-05-26

**Authors:** Sulaiman O. Aljaloud, Salam A. Ibrahim

**Affiliations:** ^1^Department of Exercise Physiology, College of Sport Sciences and Physical Activity, King Saud University, Riyadh, Saudi Arabia; ^2^Food Microbiology Laboratory, North Carolina Agricultural and Technical State University, Greensboro, NC, USA

## Abstract

The objective of this study was to understand the usage patterns of dietary supplements among professional athletes in Saudi Arabia. The survey consisted of sixteen questions divided into four categories: use of supplements, reason for consumption of supplements, personal beliefs about supplements, and behavior. The questionnaires were given to the three teams residing in Riyadh: Al Hilal, Al Nasr, and Al-Shabab. Out of the 105 athletes surveyed, we found that only 98 are currently taking dietary supplements and the mean age and standard deviation were 25.74 ± 2.90. The survey results showed a high percentage of athletes (93.3%; *n* = 98) using different dietary supplements throughout the season, 43.8% (*n* = 43) reported using supplements for performance, and 32.6% (*n* = 32) believed in health benefits as a reason for using dietary supplements. Our results showed that a total of 87 (88.7%), 81 (82.6%), and 51 (52.0%) athletes are consuming sports drinks, vitamin C, and multivitamins, respectively. Meanwhile, those supplements ranking among the least used included omega 6 (18.6%), creatine (16.3%), and Ginkgo biloba (10.2%). A majority of athletes indicated that their use of supplements was for the purpose of improving their health and performance.

## 1. Introduction

Dietary supplements in the United States, as defined by the Dietary supplement Health and Education Act of 1994, are defined as any “product” (other than tobacco) intended to supplement the diet that contains one or more dietary ingredients [1]. Dietary supplements include vitamins, minerals, herbs, meal supplements, sports nutrition products, natural food supplements, and other related products used to boost the nutritional content of the diet [2, 3]. Many athletes use different dietary supplements for a variety of reasons. Among the most popular products are ergogenic aids such as sports drinks, minerals, caffeine, Coenzyme Q10, and creatine to enhance physical performance and to tolerate pain [4]. This excess has been known to lead to serious health consequences [5]. Some supplements have even been implicated as the cause of death and disability when used improperly [6]. Therefore, the amount of dietary supplements consumed should be within the recommended range of protein, carbohydrates, and lipids for that particular product [7].

There are many athletes who use different dietary supplements for a variety of reasons. Bianco et al. [8] reported a correlation between greater knowledge of dietary supplements and reduced consumption. The ratio between dietary supplements and gender was slightly higher in male athletes compared to females [9]. Athletes involved in contact sports such as football had used supplements in higher amounts [6, 8], as such intensive sports require increased muscle mass and strength. Information on the use of supplements is usually provided by the athletes' coaches and doctors [10], who reported on the use of supplements among college football players and found that 42% were using dietary supplements and 36% reported using creatine. In the same study, more than 50% of the football players believed that protein supplements are necessary for muscle growth and development. Additionally, 65% of the players surveyed believed that the information coming from media sources such as television advertising, radio, books, and magazines motivated athletes to use supplements. Typically, local players and individuals influence or motivate professional athletes to consume dietary supplements. The players look up to professionals as role models. Athletes carry a lot of performance pressure and want to be competitive. As a result, they may misuse sports supplements to gain an advantage against opponents. Due to the lack of regulations and the increasing consumption of supplements, it is very important to understand behavioral factors that may influence the consumption of these products. Therefore, it has become necessary to educate athletes by providing better information about the risks and benefits of consuming dietary supplements. To reduce the risks from the improper use of supplements, physicians, coaches, athletic trainers, parents, health educators, and other sports professionals who have a stake in the health of these professional athletes should inform supplement users about unproven results and provide warnings about the potential harm of such dietary supplements [11, 12]. Our daily meals are not enough to boost our strength in intense sports activities. Sports supplements can provide an easy way to improve our health and build and maintain muscle mass, endurance, and power. Using carbohydrate diets containing electrolytes can hydrate the body during sporting events. Injury prevention and enhanced recovery are important benefits of using sports nutrition supplements [13]. 

Saudi Arabia supervises 153 football clubs. These clubs include various grades and age groups, which range from 12 years of age to 15, to adult teams. The main objectives of the Saudi football leagues are to promote a spirit of competition and to promote a strong base for the sport in Saudi Arabia. The Saudi Professional League (SPL) is the primary football organization in Saudi Arabia. Despite being active in sports activities, no previous survey studies have been conducted by the SPL to know whether the players are receiving accurate information regarding the use of dietary supplements. Since supplements are an integral part of players' lives, they must use these supplements properly in order to avoid negative health consequences. Therefore, the objective of this study was to understand the usage of and beliefs about dietary supplements among professional athletes in Saudi Arabia through multiple choice survey questionnaires. In addition, factors influencing the use of dietary supplements by professional athletes were described.

## 2. Methods

The Institutional Review Board (IRB) of North Carolina Agricultural and Technical State University approved this study and questionnaire. The survey method was discussed with professional athletes in the capital city of Saudi Arabia, Riyadh. The questionnaires were designed to understand perceptions of supplements among professional athletes in Saudi Arabia. Questionnaires were developed based on four categories: use of supplements, reason for consumption, personal beliefs, and behavior. A consent form was placed on top of the survey for the sports and health department policy on using human subjects along with a description of subject's specifications and the nature of the survey.

There are different ways to establish validity of an instrument; in our current study we validate the survey using different approaches: (1) we had a scientific team in our group that reviewed the survey and provided feedback; (2) we asked experts (outside the group) in the subject matter to review the items and sought their opinion and input in developing/improving the items to ensure that we were measuring what we intended to measure; and (3) we compared our survey and results with the results of similar work done in the past.

### 2.1. Sampling Method

One hundred five professional football players were recruited as subjects from three different sports teams (Al Hilal, Al Nasr, and Al-Shabab) in Riyadh, Saudi Arabia. Three different football teams in Saudi Arabia's capital of Riyadh were selected to provide the necessary data for this study. We also chose these football teams based on their location in Riyadh and their willingness to participate in this study. During the initial study, we found that each team had at least 45 players. In order to make our study statistically valid and representative, we needed at least 10% of the total team members. Therefore, this study included a total of 105 professional football players. All subjects were professional football players between the ages of 20 and 30 who were either Saudi or foreign nationals. Each team was assigned a coordinator who had the role of organizing recruitment efforts. The recruitment process included advertisement of the study via word of mouth, E-mail, posters, and announcements at weekly training meetings. The date and location of the survey meeting were communicated in the recruitment information. All methods and materials used in this study were approved by the “The Saudi Professional League” of Saudi Arabia Riyadh Human [14].

### 2.2. Translation of Questionnaire

The questionnaire was originally developed in English. However, most of the participants in this study did not have the ability to read the English version. Therefore, an Arabic version of the questionnaire was developed. Dr. Sami Siraj, who is the Assistant Professor at Taibah University in Saudi Arabia, and Dr. Osman H. Hassan, research scientist at the University of North Carolina Agricultural and Technical State University, reviewed the Arabic version of the questionnaire.

### 2.3. Survey Questionnaire

A questionnaire was developed to collect data per the objective. The questionnaire consisted of 16 questions divided into the following four categories: use of supplements, reason for consumption, personal beliefs, and behavior. Overall, the survey questions pertained to the frequency of supplement purchases as well as factors that might be considered by professional athletes when purchasing these supplements. Factors for the latter set of questions included cost/serving, cost/g of supplement, total cost of the supplement, taste, perceived results, ease of use, grams of supplement/serving, and other nutritional content such as protein (e.g., whey, casein, egg, or soy) or other forms of proteins, fats, amino acids profile, and carbohydrates. The survey also sought to determine the subjects' primary sources of information about supplements, such as magazine advertisements, an athletic trainer, strength and conditioning coach, nutritionist/dietitian, internet website, friend, family member, or health food store employee.

### 2.4. Data Collection (Survey Administration)

The researcher traveled to Riyadh, Saudi Arabia, to collect the data for this study. This study took approximately three months to administer the surveys and collect the data from the participants. During the summer of 2012, the survey was given to each sports team. The time and location for each survey were arranged and announced at least two weeks in advance. All professional players attended and participated in the survey that took place in the club classroom at 9:00 am on Monday Saudi Arabian time. Approximately 10 minutes were needed to distribute surveys and provide information, while it took about 45–50 minutes to complete the survey questionnaires. Paper-based multiple choice questionnaires were administered. 

### 2.5. Statistical Analysis

The SAS System program (SAS Inc., Cary, NC, USA) was used to compute the data for this study. The dependent variable attitudes were measured by administering the questionnaire that consisted of 16 questions divided into the four previously mentioned categories. The independent variables were the professional athletes. The first and second research questions were analyzed using chi-squared tests with a significance level of 0.05.

## 3. Results

This study focused on the use of dietary supplements by professional athletes in Saudi Arabia and those athletes' attitudes toward these supplements.  Table 1 shows the number of players from each team that participated in the survey. The teams consisted of male football players from Riyadh with ages ranging from 20 to 30 years of age. The number of players from Al Hilal was 42% (*n* = 44) followed by Al-Shabab with 31.5% (*n* = 33) players and Al Nasar with 26.5% (*n* = 28) players. The survey contained sixteen questions ranging from frequency of use of supplements to personal information such as age and level of education.

Table 2 lists the survey questions related to the reason why each athlete used dietary supplements. In the first question, we asked if the professional players involved in this study took dietary supplements. Of the 105 athletes surveyed, we found that only 98 athletes were currently taking dietary supplements (mean age and standard deviation were 25.74 ± 2.90). Thus, we decided to focus on those 98 athletes who were taking dietary supplements and eliminated the other seven. Responses showed that a high percentage of athletes (93.3%) were using different dietary supplements throughout the season.

In the question relating to the main reason for using dietary supplements, results showed a high percentage of athletes 93.3% (*n* = 98) used different dietary supplements for different reasons. For example, 43 athletes (43.8%) reported using supplements for performance, whereas 32.6% (*n* = 32) believed that improvement in health was a reason for using dietary supplements. Sixty-four athletes (65.3%) reported buying supplements from trainers or physicians, and less than 5.1% (*n* = 5) reported purchasing supplements from online stores or from other sources. Similarly, most athletes (*n* = 45; 45.9%) reported a physician as their main source of information provider on dietary supplements, followed by nutritionist (*n* = 28; 28.5%) and coach (*n* = 11; 11.2%). However, less than 10% athletes reported their sources of information as journals, magazines, and online resources.

Of the total of 98 participants who take supplements, all were males, 43.8% (*n* = 43) were under the age of 25, and most of the players in survey (*n* = 55; 56.1%) were older than 26. Forty-seven (47.9%) had a high school degree, followed by 23 (23.4%) who had some college degree, 13 (13.2%) who held a bachelor's degree, 8 (8.1%) with less than high school degree, and approximately 7 (7.1%) had a graduate or professional degree. Most of these players (*n* = 80; 81.3%) had been involved in the sport for less than nine years.

Table 3 lists the questions regarding the perception of dietary supplements and usage among professional athletes in Saudi Arabia. Questions from 8 to 15 in the study dealt with athletes' perceptions regarding the use of supplements. A majority of athletes (82.6%; *n* = 81) agreed that supplements were a healthy choice, and 67.3% (*n* = 66) believed that supplements improved endurance.

Regarding the safety of dietary supplements, most (*n* = 44; 44.9%) somewhat agreed that the supplements they used were safe, while 41.8% (*n* = 41) believed that supplements are a safe product in general. Sixty-six athletes (67.3%) agreed that supplements were a good source of energy, and 44 athletes (44.9%) supported longer training sessions. Similarly, 59.1% (*n* = 58) reported that dietary supplements increased their strength. A majority of players (*n* = 30; 30.6%) indicated that supplements were taken as a pain reliever during training sessions, and 42 players (42.8%) used supplements to enhance concentration during the game.

 In our study, we were also interested in knowing more about the types of supplements that were being used among these athletes.  Table 4 shows the list of supplements that athletes reported during the session. Athletes used a total of 23 different products. Our results showed that sports drinks were the most popular supplement used among the athletes (*n* = 87; 88.7%), followed by vitamin C (*n* = 81; 82.6%), calcium (*n* = 67; 68.3%), health bars (*n* = 58; 59.1%), and multivitamins (*n* = 51; 52.0%). Meanwhile, those ranking among the least used included omega 6 fatty acid (*n* = 18; 18.3%), creatine (*n* = 16; 16.3%), and Ginkgo biloba (*n* = 10; 10.3%).

## 4. Discussion

Professional football has been popular in Saudi Arabia. Improvement in players' performance is closely scrutinized. These players are always under pressure to perform well, win the match, and to be part of the national team. Elite athletes are using dietary supplements as a legal means to ensure a competitive edge. Each year new supplements are appearing in growing Saudi Arabia markets and intake of supplements among athletes is also increasing. It is important to be mindful of the use of supplements by professional athletes. There is little published information regarding the consumption of dietary supplements in Saudi Arabia. To the best of our knowledge, information on availability and use of supplements among professional athletes in Saudi Arabia has never been made available. There is an inherent need to increase the knowledge of dietary supplement use among consumers.

Nutritional supplements can be grouped into four basic categories: sports foods, dietary supplements, ergogenic aids, and herbs/traditional products [9, 15]. Some commonly used supplements in this category are shown in Table 4 [9]. The use of specific nutritional supplements among athletes ranges from 46% to 100% [9]. This wide variation can be attributed to different methodological differences, purpose of use, and mode of data collection. Our study shows that the prevalence of use of dietary supplements among professional athletes in Saudi Arabia is similar to nutritional supplement use among university athletes in Singapore [9]. From this study, it is believed that over 90% of the athletes in Saudi Arabia use dietary supplements, which is 10% higher compared to the study conducted among German athletes by Braun et al. [16]. Similar results were also reported by Murphy and Jeanes [17]. Authors have also reported the need of nutritional knowledge to improve the quality of performance. 

In our study, the most popular products were sports drinks and vitamin/mineral supplements, which is similar to the Singapore study. The use of sports drinks in our study is close to that shown in the study earlier reported [18, 19]. Seventy-three percent of the athletes reported using energy drinks, and 75% reported using calorie replacement products including drinks, bars, and powders [20]. Similarly, the use of vitamins and multivitamins ranges from 26 to 82% among athletes. The use of multivitamins and vitamin C is higher than 50% and 80%, respectively [21, 22]. The reasons these athletes use vitamins are primarily to stay healthy and to prevent illnesses during the game season [23].

Similarly, there has been a study involving German athletes where the intake of sports drink was lower (69%) when compared to the intake percentage (87%) of our study. Likewise, those athletes who took vitamins are comprised of 81% of the Saudi athletes while Germans lagged behind at 76%. However, when it came to mineral intake, Germans consumed 87% while Saudi athletes consumed 60%. Carbohydrates ranked evenly with Germans taking in 64% compared to the Saudi's 67% [16].

We also found in our study that the use of other dietary supplements such as ephedra, omega 6, creatine, and Ginkgo biloba was lower. The use of the most popular supplement, creatine, is only 16.3%, which is very low when compared to the western countries, including the United States [24]. Jonnalagadda et al. [10] reported that 36% of athletes used creatine, which was higher compared to our study (16.3%). In recent years, the use of supplements among athletes in a number of sports has been increasing to improve performance [10]. Some of the supplements available in Saudi Arabia were also found to be contaminated with microorganisms [25–27]. Therefore, it is important to understand how these products work and whether these products provide worthwhile benefits while considering possible long-term health effects.

Of the 105 athletes surveyed, we found that 93.3% (98) are currently taking dietary supplements. Survey results showed that a majority of athletes use different dietary supplements throughout the season either to enhance performance or simply to improve their health. In earlier studies [24], it has been noted that the use of supplements among male athletes is primarily to enhance performance. Vitamin and mineral supplements are often used by athletes as ergogenic aids to improve performance, as described by [28].

 Nutritional supplements can also play an important role in helping athletes consume the correct amount of calories, carbohydrates, and proteins in their diet. However, inappropriate use or contamination may cause health problems [9].

We found that most of the athletes had received their information about supplements either from physicians or nutritionists. A total of 45 surveyed athletes of 98 (45.9%) received the information from their physicians, which is similar to the study conducted by Braun et al. [16].

According to a study by Froiland et al. [18], male athletes were more likely to obtain information about the use of supplements from a store nutritionist, fellow athletes, friends, or from a coach. These results were similar to ours. Similarly, in a study [19], health professionals and the Internet were the most common information sources, while friends and colleagues often recommended the use of supplements. Regular use of supplements among athletes would thus indicate that a regular diet alone is not sufficient to provide the necessary nutrients and energy during sports performance. Sports such as football rely primarily on a combination of the phosphagen system, anaerobic glycolysis, and aerobic metabolism [19]. To enhance performance and strength and for the overall improvement of health, it is important to meet all these metabolic demands by taking different supplements. Therefore, athletes should be better informed and educated about the benefits associated with the consumption of supplements.

## 5. Conclusions

Our results showed that a total of 88 (88.7%), 81 (82.6%), and 51 (52.0%) athletes were consuming sports drinks, vitamin C, and multivitamins, respectively. Meanwhile, those supplements ranking among the least used included omega 6 (18.6%), creatine (16.3%), and Ginkgo biloba (10.2%). Our study showed that the use of dietary supplements varies with each individual professional athlete for several reasons. According to the given survey, when asked about their use of supplements and their reasons for consumption, a majority of athletes expressed their desire to improve health and performance. 

## Figures and Tables

**Table 1:** Teams that participated in the survey.

Team name	Participants	
	%	*n*
(1) Al Hilal	41.9	44
(2) Al Nasr	26.5	28
(3) Al-Shabab	31.5	33

Total	100.0	105

**Table 2:** The usage of dietary supplements among professional athletes (n = 105).

Response	%	*n*
(1) Do you currently take dietary supplements?		
No	6.6	7
Yes	93.3	98
(2) What is the main reason of using dietary supplements?		
Improve my health	32.6	32
Prevent injury	9.1	9
Recovery	3.0	3
Physical appearance	11.2	11
Performance	43.8	43
(3) Where do you usually buy your dietary supplements?		
Retail store or pharmacy	11.2	11
Athletic trainer or physician	65.3	64
Nutritionist or dietician	18.3	18
Online store	4.0	4
Other	1.0	1
(4) From whom do you get information about dietary supplements?		
Coach	11.2	11
Physician	45.9	45
Nutritionist or dietician	28.5	28
Academic journals	4.0	4
Online	8.1	8
Magazine	2.0	2
(5) Age		
<25 years old	43.8	43
>26 years old	56.1	55
(6) What is your highest level of education?		
Less than high school	8.1	8
High school	47.9	47
Some college	23.4	23
Bachelor's degree	13.2	13
Graduate or professional degree	7.1	7
(7) How long have you been a professional football player?		
<9 years	81.6	80
>10 years	18.3	18

These results showed that the response does differ significantly (*P* < 0.0001) from the hypothesized value (0.05) indicating that reasons for taking dietary supplements differ from each individual.

**Table 3:** Perception of dietary supplements and usages among professional athletes (*n* = 98).

Response	%	*n *
(8) Dietary supplements make me healthier		
Agree	82.6	81
Somewhat agree	13.2	13
Neither agree nor disagree	4.0	4
(9) Dietary supplements improve my endurance		
Agree	67.3	66
Somewhat agree	16.3	16
Neither agree nor disagree	12.2	12
Somewhat disagree	4.0	4
(10) Dietary supplements are safe to use		
Agree	41.8	41
Somewhat agree	44.9	44
Neither agree nor disagree	8.1	8
Somewhat disagree	3.0	3
Don not know	2.0	2
(11) Dietary supplements provide me with more energy		
Agree	67.3	66
Somewhat agree	25.5	25
Neither agree nor disagree	6.1	6
Somewhat disagree	1.0	1
(12) Dietary supplements increase the amount of training I can undergo		
Agree	44.9	44
Somewhat agree	32.9	32
Neither agree nor disagree	20.4	20
Somewhat disagree	1.0	1
Disagree	1.0	1
(13) Dietary supplements increase my strength		
Agree	59.1	58
Somewhat agree	21.4	21
Neither agree nor disagree	16.3	16
Somewhat disagree	3.0	3
(14) Dietary supplements increase my ability to cope with pain		
Agree	30.6	30
Somewhat agree	31.4	31
Neither agree nor disagree	28.5	28
Somewhat disagree	7.1	7
Disagree	2.0	2
(15) Dietary supplements improve my concentration		
Agree	42.8	42
Somewhat agree	23.4	23
Neither agree nor disagree	25.5	25
Somewhat disagree	5.1	5
Disagree	2.0	2

Note. These results showed that the response does differ significantly (*P* < 0.0001) from the hypothesized value (0.05) indicating that reasons for taking dietary supplements differ for each individual.

**Table 4:** Type of dietary supplements and frequency of use among professional athletes (*n* = 98).

Category of supplements	No	Yes
	(%) *n*	(%) *n*
Sport supplements		
Sport drinks	(11.2) 11	(88.7) 87
Health bar	(40.8) 40	(59.1) 58
Red bull energy drink	(74.4) 73	(25.5) 25
Vitamins		
Vitamin C	(17.3) 17	(82.6) 81
Vitamin D	(56.1) 55	(43.8) 43
Vitamin E	(73.4) 72	(26.5) 26
Vitamin B	(59.1) 58	(40.8) 40
Multivitamin	(47.9) 47	(52.0) 51
Minerals		
Iron supplement	(42.8) 42	(57.1) 56
Calcium	(31.6) 31	(68.3) 67
Carbohydrate		
Carbohydrate electrolyte beverage	(31.6) 31	(68.3) 67
Fructose syrup	(85.7) 84	(14.2) 14
Protein		
Amino acids	(73.4) 72	(26.5) 26
Ephedra	(86.7) 85	(13.2) 13
Weight gainers	(77.5) 76	(22.4) 22
Fish oils		
Omega 3	(54.0) 53	(45.9) 45
Omega 6	(81.6) 80	(18.3) 18
Herbals		
Ginseng	(71.4) 70	(28.5) 28
Gingko biloba	(89.7) 88	(10.3) 10
Ergogenic aids		
Slimming products	(71.4) 70	(28.5) 28
Coenzyme	(76.5) 75	(23.4) 23
Caffeine	(41.8) 41	(58.1) 57
Creatine	(83.6) 82	(16.3) 16

Since the *P* value is 0.0001, we can conclude that there is statistically significant difference between the frequencies of use of dietary supplements among professional athletes.
